# The Diagnostic Role of Tumor and Inflammatory Biomarkers in Ascitic Fluid: A Systematic Review

**DOI:** 10.3390/medicina61091582

**Published:** 2025-09-01

**Authors:** Gentiana Ratkoceri Hasi, Joško Osredkar, Aleš Jerin

**Affiliations:** 1 Faculty of Medicine, University of Ljubljana, 1000 Ljubljana, Slovenia; ratkocerigentiana@gmail.com; 2 Clinical Biochemistry Clinic, University Clinical Center of Kosovo, 10000 Prishtina, Kosovo; 3 Institute of Clinical Chemistry and Biochemistry, University Medical Centre Ljubljana, 1525 Ljubljana, Slovenia; josko.osredkar@kclj.si; 4 Faculty of Pharmacy, University of Ljubljana, 1000 Ljubljana, Slovenia

**Keywords:** ascitic fluid, CA125, CEA, CRP, diagnostic accuracy, IL-6, inflammatory biomarkers, malignant ascites, tumor markers, VEGF

## Abstract

*Background and Objectives*: Diagnosing the underlying cause of ascites remains complex, especially when cytology results are inconclusive. Measuring biomarkers directly in ascitic fluid may offer better diagnostic insight than serum testing alone. This review evaluated the clinical utility of tumor and inflammatory markers in ascitic fluid. *Materials and Methods*: A systematic search was conducted in PubMed and Scopus for studies published from January 2014 to December 2024, with the final search carried out in May 2025. The included studies were observational, comparative or biomarker validation studies evaluating ascitic fluid markers for diagnosing malignant and inflammatory ascites. The extracted outcomes included diagnostic accuracy metrics such as area under the curve (AUC), sensitivity and specificity. Risk of bias was evaluated using the ROBINS-I tool. Studies were excluded if they were case reports, animal studies, cytology-only analyses, or if they lacked biomarker data in ascitic or peritoneal fluid. *Results*: Forty-two studies met the inclusion criteria. CEA showed high diagnostic performance when measured in ascitic fluid. Combining markers or using ascitic-to-serum ratios improved diagnostic reliability. Inflammatory markers in ascitic fluid, such as CRP, IL-6 and VEGF added diagnostic value when cytology was inconclusive. *Discussion and Conclusions*: Evaluating biomarkers in ascitic fluid improved diagnostic accuracy. However, the included studies showed considerable methodological heterogeneity and moderate risk of bias.

## 1. Introduction

Ascites, defined as an abnormal accumulation of fluid in the peritoneal cavity, is a clinical sign commonly associated with underlying conditions, such as liver cirrhosis, cardiac dysfunction, renal impairment, infections and malignancies [1]. Although its presence is generally linked to poor prognosis [2,3], patient outcomes depend on the cause. Accurate diagnosis requires the integration of ascitic fluid analysis with clinical and pathological evaluation [4].

Traditional classifications based on total protein content in ascitic fluid have shown limited diagnostic reliability [4,5]. The introduction of the serum-ascites albumin gradient (SAAG) improved the differentiation between portal hypertension-related and non-portal causes of ascites, including peritoneal inflammation and malignancy [4]. However, when cytology is inconclusive, the SAAG alone may be insufficient for definitive diagnosis.

In recent years, tumor and inflammatory biomarkers in ascitic fluid have gained attention as supplementary tools to help determine the etiology of ascites. Tumor markers, which are molecules produced by tumor cells or by the host in response to tumor presence, are widely used in oncology for screening, diagnosis, monitoring treatment response and prognosis [6]. Their application in ascitic fluid analysis may offer additional diagnostic value when conventional methods are insufficient.

Several tumor markers, such as alpha-fetoprotein (AFP), carcinoembryonic antigen (CEA), cancer antigen (CA) 19-9 and CA 125 have been studied for their potential to differentiate between malignant and non-malignant ascites. Many reports have shown that these markers are often elevated in ascitic fluid in cases of hepatocellular carcinoma or gastrointestinal and ovarian cancers [5,7,8,9,10,11]. The proteomic analysis of ascitic fluid has also been explored as a strategy to identify novel biomarkers in hepatocellular carcinoma [12].

Some studies have demonstrated that measuring tumor markers in serum or ascitic fluid may help distinguish malignant ascites. However, small sample sizes and inconsistent marker selection have limited the strength of these findings [6]. As a result, the diagnostic value of these markers in ascites remains under debate. Inflammatory biomarkers have also been investigated for their potential to clarify the underlying cause of ascites. Markers such as C-reactive protein (CRP), vascular endothelial growth factor (VEGF), lactate dehydrogenase (LDH) and several interleukins have been studied in this context. Among these, LDH and VEGF have shown stronger associations with malignant ascites [4,6].

There remains a debate regarding whether ascitic or serum levels provide more clinically meaningful information. Ascitic fluid may better reflect local inflammation, whereas serum levels may indicate systemic processes [13]. VEGF, in particular, is often significantly elevated in malignant ascites, supporting its utility as a potential localized tumor marker [4].

This systematic review aimed to evaluate the diagnostic and prognostic performance of tumor and inflammatory markers measured in ascitic fluid, with a focus on their role in distinguishing malignant, infectious and inflammatory causes of ascites. The review addressed the following research questions:

- Do tumor markers measured in ascitic fluid offer clinically useful diagnostic and prognostic accuracy for malignant ascites?
- Are inflammatory markers in ascitic fluid effective in differentiating between inflammatory and malignant ascites?
- Does combining tumor and inflammatory markers enhance the ability to distinguish between malignant and non-malignant ascites?

Hypothesis: We hypothesized that biomarkers measured directly in ascitic fluid would demonstrate sufficient diagnostic and prognostic utility for differentiating between malignant and inflammatory ascites, due to their ability to reflect localized disease activity within the peritoneal cavity. Ongoing debate regarding the clinical utility of tumor and inflammatory markers in ascitic fluid supports the need to evaluate their diagnostic performance in this review. Following the PRISMA (Preferred Reporting Items for Systematic Reviews and Meta-Analyses) guidelines [14], this review synthesized current evidence to evaluate the clinical value of ascitic biomarkers and support their application in diagnostic workflows.

## 2. Materials and Methods

This systematic review was conducted to evaluate the diagnostic and prognostic performance of tumor and inflammatory biomarkers measured in ascitic fluid, with the aim of distinguishing malignant from inflammatory causes of ascites.

### 2.1. Study Design and Eligibility Criteria

This review included observational studies (retrospective or prospective), clinical comparison studies and biomarker validation studies evaluating tumor and inflammatory markers in ascitic fluid for the diagnosis of malignant or inflammatory ascites. Studies were excluded if they involved animals, were case reports, did not measure markers in ascitic or peritoneal fluid or were published in languages other than English.

### 2.2. Information Sources

A comprehensive literature search was conducted using PubMed and Scopus databases. The search covered studies published between January 2014 and December 2024. The final search was carried out in May 2025, and reference lists of included studies were also screened to capture any additional eligible records.

### 2.3. Search Strategy

Searches used Boolean operators (AND/OR) to combine terms relating to ascitic or peritoneal fluid, tumor markers (e.g., CEA, CA125, CA19-9, HE4), inflammatory markers (e.g., CRP, IL-6, VEGF, TNF-α) and emerging biomarkers (e.g., microRNAs, exosomes, extracellular vesicle proteins). Filters were applied to limit results to human studies, English-language publications and the specified time frame.

The complete search strategy, including all terms and combinations used are shown in Appendix A.

### 2.4. Study Selection

**Figure 1:**
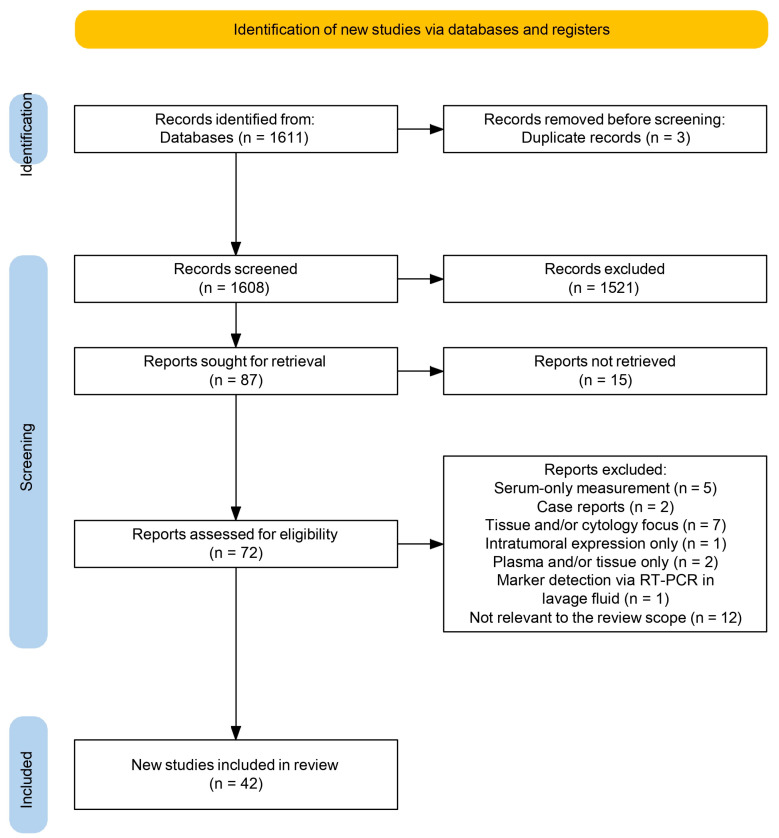
PRISMA 2020 flow diagram of literature selection process. The figure was created using the official PRISMA 2020 flow diagram tool available at www.prisma-statement.org (accessed on 5 August 2025) [14].

Reviewers conducted an initial screening of titles and abstracts to determine the preliminary eligibility of studies. Full-text screening was then performed for the selected studies. To minimize selection bias, a random subset of included and excluded studies were independently re-evaluated. Any uncertainties or discrepancies were resolved by consulting a senior researcher or by referring to the predefined inclusion criteria. The study selection process was documented using the PRISMA 2020 flow diagram (Figure 1), created using the official tool available at www.prisma-statement.org [14].

Findings from the eligible studies were synthesized narratively. A formal meta-analysis of diagnostic accuracy was not performed because the included studies varied substantially in design (retrospective vs. prospective and case–control), patient selection and disease spectrum, assay platforms and cutoff thresholds. These methodological differences precluded valid pooling of sensitivity/specificity or AUC across studies. Instead, results are presented as study-level estimates and typical ranges to facilitate transparent comparison.

### 2.5. Data Collection and Extraction

Data from the included studies were extracted using a standardized form. The following information was collected:

- Study characteristics (author, year, country, sample size, age, duration and setting);
- Type of marker(s) evaluated (tumor and inflammatory);
- Fluid analyzed (ascitic fluid);
- Diagnostic performance (sensitivity, specificity and AUC, if available);
- Prognostic significance (if available);
- Study design (e.g., prospective, retrospective).

Risk of bias for all 42 included studies was evaluated using the ROBINS-I framework. A traffic light plot was generated using the robvis web application developed by McGuinness and Higgins [15], providing a visual summary of bias across each domain.

### 2.6. Summary Measures

Diagnostic performance was summarized using standard measures, such as sensitivity, specificity and area under the ROC curve (AUC). When available, diagnostic odds were also reported.

### 2.7. Synthesis of Results

Due to methodological heterogeneity and varying diagnostic endpoints, a narrative synthesis of results was performed. Data were organized into thematic tables, classified by marker type (tumor markers or inflammatory markers). A meta-analysis was considered but not performed due to heterogeneity across study design and reporting metrics. Visual summaries, including performance tables and heatmaps, were constructed to illustrate diagnostic performance across different marker categories and fluid types.

### 2.8. Reporting Standards and Ethical Statement

This systematic review was conducted in accordance with the PRISMA 2020 guidelines for reporting a systematic review [14]. The review was not prospectively registered. Ethical approval was not required, as this study involved secondary analysis of previously published data. Generative AI tools were used in a limited capacity to assist with data interpretation and figure formatting under direct author supervision.

## 3. Results

### 3.1. Study Selection and Overview

The systematic search identified a total of 1611 records across all databases. After duplicate removal, 1608 articles remained for title and abstract screening. Of these, 72 full-text articles were assessed for eligibility. In total, 42 studies met the inclusion criteria and were included in the final analysis. The screening process is illustrated in Figure 1 (PRISMA flow diagram). Full-text articles were excluded due to insufficient data on ascitic or serum tumor marker levels, exclusive focus on tissue or cytology, case report format or lack of relevance to the review scope.

Following a comprehensive screening process, 42 studies were included in this review (Table 1). The included studies were heterogeneous in design, population and laboratory methodology, which limited the feasibility of quantitative synthesis and made direct comparison between studies difficult. These studies were geographically diverse and represented a range of clinical settings, including oncology centers, academic hospitals and multicenter consortia. Sample sizes varied from 22 to over 1000 patients, with most study populations ranging in age from 50 and 70 years. Most studies focused on adults with suspected malignant, infectious or inflammatory ascites. Prospective observational studies were among the most common, even though several high-quality retrospective cohorts and diagnostic validation studies were also included. This variation in study design and setting increases the breadth of the evidence base but also adds complexity when interpreting pooled diagnostic performance.

### 3.2. Risk-of-Bias Assessment

**Figure 2:**
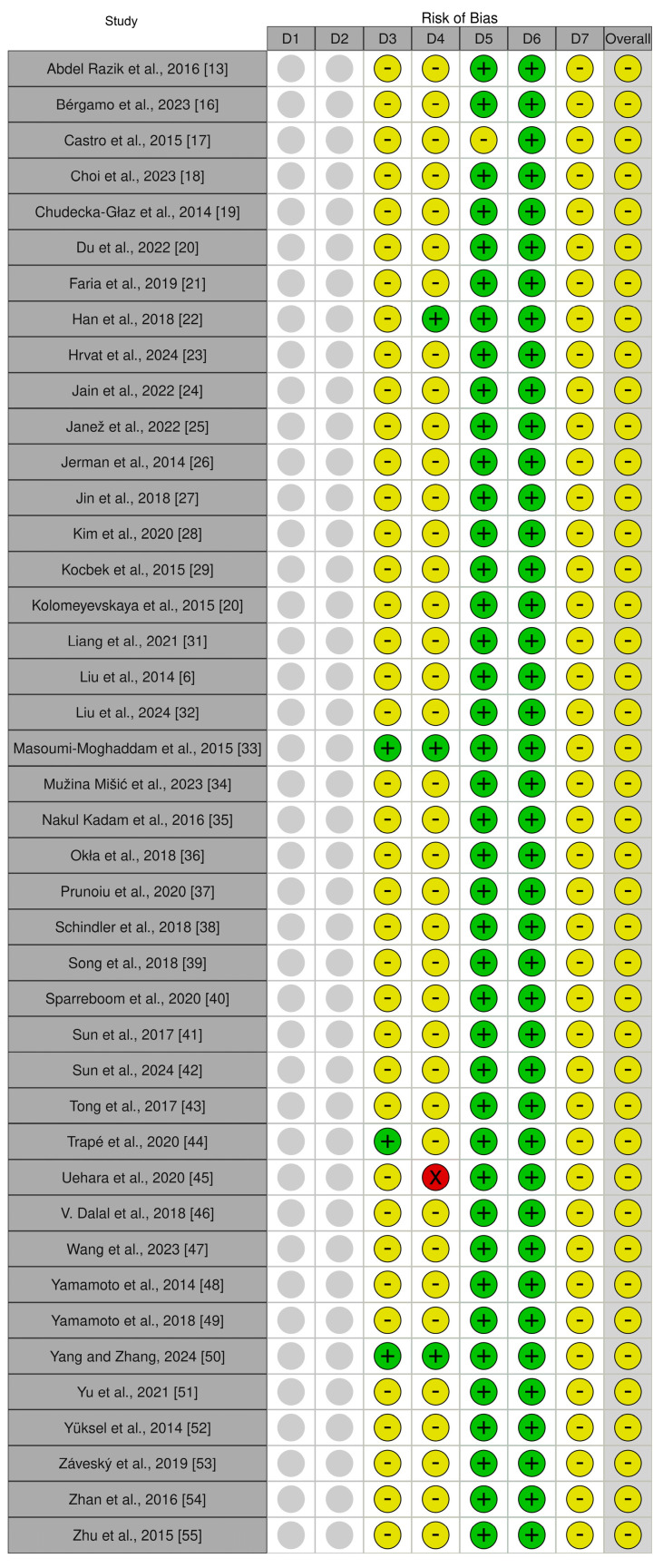
Traffic light plot summarizing ROBINS-I assessment. The figure was created using the robvis tool [15]. D1: random sequence generation; D2: allocation concealment; D3: blinding of participants and personnel; D4: blinding of outcome assessment; D5: incomplete outcome data; D6: selective reporting; D7: other sources of bias. Judgement: red—high, yellow—unclear, green—low, grey—not applicable [6,13,16,17,18,19,20,21,22,23,24,25,26,27,28,29,31,32,33,34,35,36,37,38,39,40,41,42,43,44,45,46,47,48,49,50,51,52,53,54,55].

Risk of bias across the included studies was evaluated using the ROBINS-I tool [15]. The overall judgments were presented in a corresponding traffic light plot (Figure 2).

Across the 42 included studies, the overall methodological quality was acceptable, with none rated as high risk of bias. However, several domains consistently raised concerns. Blinding was rarely feasible, as ascitic fluid was collected in clinical contexts where the underlying diagnosis was already suspected; accordingly, blinding of participants and personnel was judged unclear in 39 studies, and outcome assessment was judged unclear in 38 and high in one. Retrospective designs, which comprised much of the evidence base, further limited standardized follow-up and increased susceptibility to spectrum bias, particularly in small single-center or case–control cohorts. In contrast, incomplete outcome data and selective reporting posed little risk, with 41 studies rated low for completeness, and all judged low for reporting transparency. Random sequence generation and allocation concealment were not applicable to the observational designs. Overall, these patterns explain why all studies were classified as having “some concerns”, reflecting limitations in design and blinding rather than systematic flaws in reporting or laboratory methods.

### 3.3. Biomarker Evaluation in Ascitic Fluid

**Figure 3:**
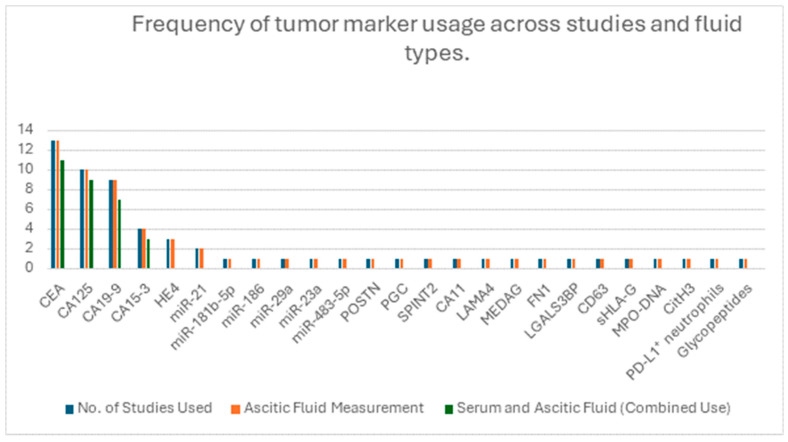
Frequency of tumor marker evaluation across included studies. The chart presents the number of studies that measured each marker in ascitic fluid or used it in combination, such as ascitic-to-serum ratios or multi-marker panels. Markers are listed in descending order based on total frequency of use.

**Figure 4:**
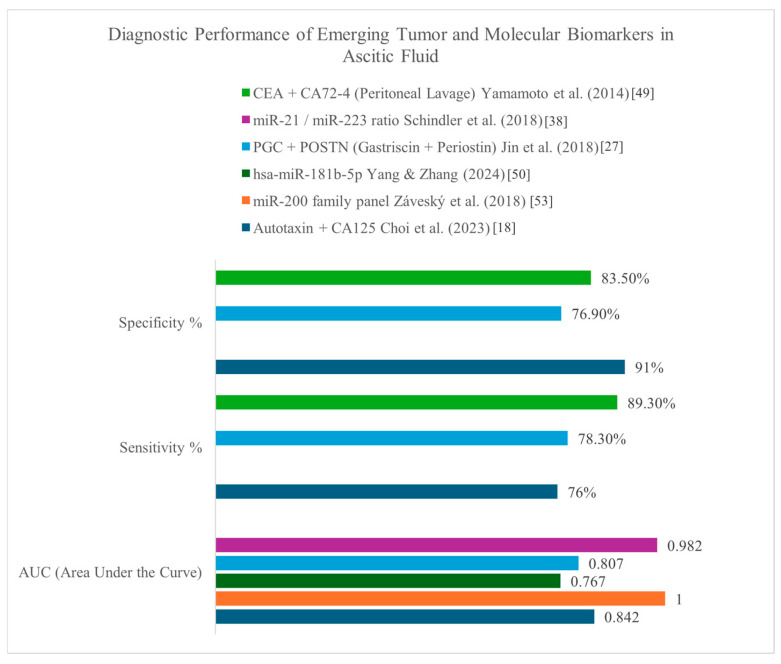
Sensitivity, specificity and AUC values for selected biomarkers and biomarker combinations measured in ascitic or peritoneal fluid. Data were extracted from studies included in the systematic review. AUC values are presented for individual markers and miRNA panels. Sensitivity and specificity are reported when available [18,27,38,49,50,53].

Tumor markers were analyzed in a range of biological fluids as presented in Table 2, most commonly in ascitic fluid, though several studies also included peritoneal or pleural fluid. Among the 30 studies that investigated tumor markers, the ones most frequently measured were CEA, CA125 and CA 19-9, while less commonly investigated markers included HE4, CA72-4, CA 15-3 and CYFRA 21-1 (Figure 3). A few studies explored novel candidates such as autotaxin (ATX) and microRNAs. Sensitivity, specificity and AUC values for selected biomarkers and biomarker combinations is presented in Figure 4. CEA was generally among the best-performing single ascitic markers, with accuracy varying by tumor type and cohort. Its value was most evident in cancer-specific cohorts, such as colorectal cancer, where Song et al. [39] reported an AUC of 0.996 for ascitic CEA. High accuracy was also observed in mixed malignancy cohorts when incorporated into diagnostic algorithms [20,32]. In broader heterogenous populations, single-marker CEA performance was more modest (AUCs generally ~0.78–0.86), but its utility increased substantially in combination with other markers, such as CA19-9 and CA125, or when applied in ascitic-to-serum ratio models. These multi-marker approaches frequently achieved sensitivities and specificities above 85–95%, underscoring the added value of integrated strategies over single-marker testing.

CA 125 and CA 19-9 showed diagnostic utility when interpreted together or used in ratio-based models. In patients with pseudomyxoma peritonei, elevated tumor marker counts (including CA125, CA19-9, and CEA) were linked to poorer surgical outcomes and reduced survival [31,47]. Among the less-frequently studied markers, HE4 showed high specificity though with limited sensitivity, ATX was associated with advanced-stage ovarian cancer and reduced disease-free survival, with one study reporting an AUC of 0.842 [18], while a microRNA ratio (miR-21/miR-223) achieved an AUC of 0.982 for differentiating malignant from non-malignant ascites [38]. While not all studies assessed prognosis, several found that higher biomarker levels were associated with advanced disease or poorer clinical outcomes [20,30], indicating potential roles beyond diagnosis in treatment stratification and prognostic assessment.

**Figure 5:**
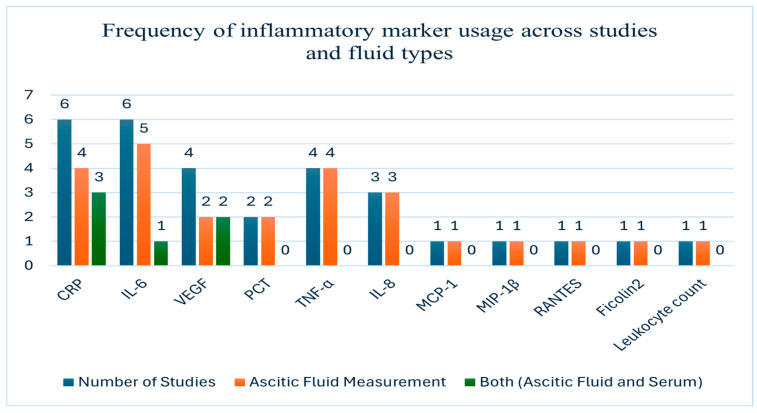
Frequency of inflammatory marker usage across studies and fluid. The chart illustrates how often each marker was evaluated, distinguishing between ascitic fluid measurement or ascitic fluid and serum together. Sample sizes across the included studies ranged from 22 to 1012 patients. Abbreviations: CRP—C-reactive protein; IL—interleukin; VEGF—vascular endothelial growth factor; TNF—tumor necrosis factor; MCP—monocyte chemoattractant protein; MIP—macrophage inflammatory protein; RANTES—regulated on activation, normal T-cell expressed and secreted; PCT—procalcitonin.

Twelve studies assessed inflammatory markers in ascitic or peritoneal fluid, with many also comparing them to serum levels (Table 3). Frequency of inflammatory marker usage across studies is presented in Figure 5. The most frequently assessed were C-reactive protein (CRP), interleukin-6 (IL-6), tumor necrosis factor-alpha (TNF-α) and vascular endothelial growth factor (VEGF), either alone or as part of diagnostic panels.

CRP showed consistent diagnostic value; for example, Abdel-Razik et al. [13] reported AUCs of 0.842 in ascitic fluid and 0.821 in serum, with the highest accuracy when combined. Yüksel et al. [52], likewise, found ascitic CRP to be superior to serum for distinguishing malignant from benign ascites. In postoperative settings, elevated peritoneal CRP predicted early anastomotic leakage [34] though its role in malignancy remains limited. IL-6 demonstrated strong diagnostic and prognostic potential, reaching an AUC of 1.000 in ovarian cancer when used alone or with VEGF-A [46] and was associated with poorer survival when elevated alongside TNF-α [30]. VEGF levels were consistently higher in malignant ascites, with reported AUCs ranging from 0.94 to 1.000 across multiple studies, while serum VEGF also showed diagnostic utility (AUC 0.921) [13]. Less frequently assessed markers also offered insight: MMP-9 in combination with CRP aided in detecting postoperative complications [34], whereas the VEGF levels in ascitic fluid were associated with disease progression and stage in ovarian cancer [31].

Overall, inflammatory markers provided the greatest diagnostic value when used in combination, reflecting local immune and vascular changes and complementing tumor markers in differentiating malignant ascites from infectious or reactive causes.

### 3.4. Diagnostic Algorithm and Interpretation

**Figure 6:**
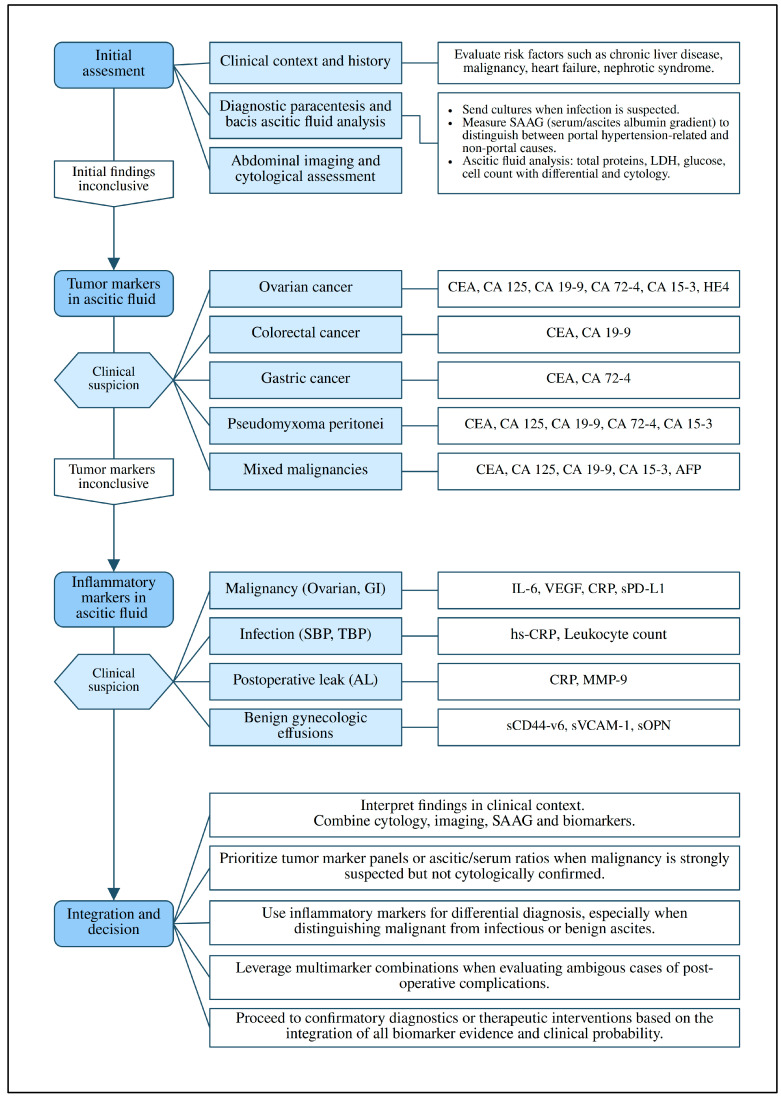
Diagnostic algorithm for ascitic fluid evaluation. The algorithm integrates cytology, imaging and ascitic biomarkers. When cytology is negative or inconclusive, tumor markers (CEA, CA125, CA19-9, CA72-4, HE4 and AFP) and ascitic-to-serum ratios improve diagnostic accuracy, while inflammatory markers (IL-6, VEGF, CRP and MMP-9) support differentiation of malignancy from infection or benign effusions and predict postoperative complications. Notes: CYFRA21-1 has been mainly reported in gastric cancer [49] and required further validation. VCAM1 and CD146 are exploratory markers in benign gynecologic effusions and should be considered adjunctive rather than primary diagnostic tools.

A diagnostic algorithm was developed to summarize the main findings of this review into a clinically applicable framework (Figure 6). It presented a structured approach to evaluating ascites, beginning with fluid collection and standard biochemical analysis. Based on the suspected underlying cause, selected tumor or inflammatory markers could then be applied to improve diagnostic accuracy.

**Figure 7:**
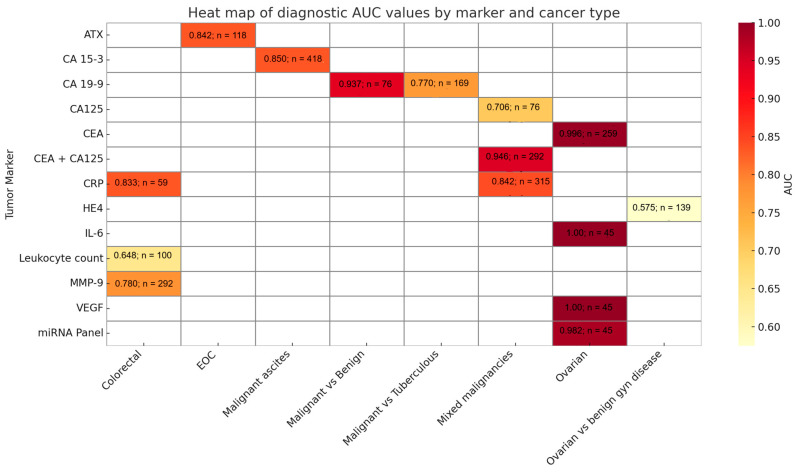
Heatmap of diagnostic AUC values and sample sizes for tumor and inflammatory markers across different cancer types. Each cell represents the reported area under the curve (AUC) for a biomarker–cancer combination, with color intensity corresponding to diagnostic accuracy. Sample sizes across studies included in this review ranged from 22 to 1012 patients. Abbreviations: AUC—area under the curve; EOC—epithelial ovarian cancer; “Ovarian vs. benign gyn disease”—comparison between malignant ovarian cancer and benign gynecologic conditions (e.g., endometriosis); “Mixed malignancies”—includes patient cohorts with multiple cancer types; “Leukocyte count”—total WBC in ascitic fluid; “CEA + CA125”—combined marker model [13,16,19,20,32,34,37,38,39,40,43,46,55].

Although ascitic fluid was preferred due to its proximity to peritoneal pathology, alternative sampling methods such as peritoneal washings were considered in patients without free fluid. Some studies also examined the use of composite indices or statistical models, which suggested added diagnostic value in selected cases. Figure 7 supports the algorithm by illustrating marker performance and cancer-type comparisons using AUC-based visual summaries.

## 4. Discussion

### 4.1. Summary of Main Findings

This systematic review followed PRISMA guidelines and aimed to evaluate the diagnostic and prognostic value of tumor and inflammatory markers in ascitic and related peritoneal fluids. A total of 1611 records were identified through searches across major databases. After removing duplicates and completing title, abstract and full-text screening, 42 studies met the inclusion criteria (Figure 1). Studies were excluded for focusing on serum-only marker measurements, tissue or cytology-based findings, case reports, plasma-only analyses or for using non-standard methods such as reverse transcription polymerase chain reaction (RT-PCR) on lavage samples. One exception was the study by Massoumi Moghaddam et al. [33], which examined IL-6 in tumor tissue, but was included due to its relevance in predicting post-treatment ascites. The included studies were conducted in oncology centers [18,20,38] and academic hospitals [36,48] and used a variety of designs including prospective, retrospective and cross-sectional formats. Sample sizes ranged from 22 participants in the study by Kolomeyevskaya et al. [30] to over 1000 in Trapé et al. [44]. Most study populations included participants aged between 50 and 70 years. The differences in patient groups, study designs, fluid types, markers tested and how results were reported made it especially important to assess the risk of bias in a clear and structured way, as shown in Figure 2.

Data were presented in a baseline summary table (Table 1). Many studies reported complete diagnostic metrics, including AUC, sensitivity and specificity, while others lacked full data. All relevant information was extracted when available; missing data were noted separately. Most studies assessed biomarkers in ascitic fluid. Serum measurements were included when both fluids were analyzed in the same study, as seen in Liu et al. [6], Abdel-Razik et al. [13] and Yu et al. [51]. Some studies evaluated peritoneal or pleural fluid when ascitic fluid was absent. For example, Yamamoto et al. [49] and Jain et al. [24] measured CEA and CA72-4 in intraoperative peritoneal washings, especially in early-stage gastrointestinal cancers, where cytology alone was inconclusive.

Among tumor markers, CEA, CA125 and CA19-9 were the most commonly studied. CEA consistently showed strong performance, with Song et al. [39] reporting an AUC of 0.996 in colorectal cancer and similarly high values observed in ovarian cancer by Du et al. [20] and Liu et al. [6]. These markers performed better when used in combination or when interpreted using ascitic-to-serum ratios, as demonstrated by Trapé et al. [44] and Tong et al. [43]. Figure 3 displays the frequency of tumor marker use across the included studies. Figure 4 summarizes the diagnostic performance of selected markers and marker combinations.

Twelve studies evaluated inflammatory markers such as CRP, IL-6, VEGF and TNF-α. These markers were most useful when applied to clinical decision-making, particularly in distinguishing malignant ascites from benign or infectious causes. Abdel-Razik et al. [13] found that CRP measured in ascitic fluid had higher diagnostic accuracy (AUC 0.842) compared to serum CRP (AUC 0.821). V. Dalal et al. [46] reported that IL-6 and VEGF combined reached an AUC of 1.000 in the diagnosis of ovarian cancer. Kolomeyevskaya et al. [30] associated elevated IL-6 and TNF-α levels in ascitic fluid with reduced progression-free survival. In a surgical setting, Mužina Mišić et al. [34] showed that CRP in peritoneal drain fluid predicted early anastomotic leakage. Figure 5 shows the frequency of inflammatory marker use across different fluid types. A proposed diagnostic algorithm (Figure 6) integrates these findings into a practical framework to support clinical evaluation.

Emerging markers provide additional, though preliminary, insights. HE4 offered high specificity but limited sensitivity, particularly in ovarian cancer [36]. ATX achieved an AUC of 0.842 and was linked to worse survival [18]. MicroRNA profiles such as the miR-21/miR-223 ratio reached very high accuracy (AUC 0.982) [38], while Záveský et al. [53] demonstrated the potential of MSLN in epithelial ovarian cancer. However, evidence for these markers is restricted to small, often retrospective studies without external validation. Their role remains investigational and requires prospective multicenter confirmation before integration into clinical practice.

### 4.2. Interpretation of Findings in Context

The results confirmed that ascitic fluid provided valuable diagnostic information in patients with suspected malignancy and often outperformed serum. Measurements of tumor and inflammatory markers in ascitic fluid provided strong diagnostic support across gastrointestinal and ovarian cancers. These markers performed best when interpreted in combination or through fluid-to-serum ratios [32,44]. Inflammatory markers, although less specific, added important context by reflecting local immune and vascular processes. For example, ascitic CRP showed higher diagnostic accuracy than serum CRP in differentiating malignant from benign ascites [13,52].

Cytology remains the diagnostic standard, but several studies demonstrated that tumor or inflammatory markers improved diagnostic clarity when cytologic findings were inconclusive [24,49,51]. In situations where ascites was absent, peritoneal washings or lavage fluid were also diagnostically useful, with CA72-4 and CEA measured reliably to support decision-making in early-stage disease [24,49]. This highlighted the methodological diversity across studies, where even alternative fluid sources could expand diagnostic options. Collectively, these findings indicated that ascitic fluid biomarkers complemented existing diagnostic tools and helped address gaps in clinical practice.

### 4.3. Clinical Implications

As outlined in the preceding section, ascitic biomarkers consistently demonstrate diagnostic and prognostic value that extends beyond the limitations of cytology. In daily practice, cytology is often negative or inconclusive despite a strong clinical suspicion of malignancy, creating delays in diagnosis and management. Biomarker testing proposes a practical solution in these situations.

In gastric cancer, several groups have suggested that markers such as CEA, CA19-9 and CA125 in ascitic fluid may help reveal peritoneal involvement when cytology is negative, potentially supporting earlier therapeutic decisions [39,49]. In ovarian cancer, ascitic HE4 and CA125 have been proposed as useful adjuncts when cytology is inconclusive, with multiple studies reporting sensitivities above 80–90% [6,22,32,36,45]. Tong et al. [43] further indicated that CEA combined with CA125 could strengthen classification when cytology was inconclusive. Multi-marker approaches, as described by Du et al. [20], Jain et al. [24], Trapé et al. [44] and Bérgamo et al. [16] suggest that panels could provide greater confidence than cytology alone. In regions where tuberculosis complicates diagnosis, marker ratios and globulin indices have been proposed to help separate malignant from infectious ascites [51].

Inflammatory mediators may offer additional support. IL-6 and VEGF have been reported as indicators of malignant ascites [25,31], while models that included CA125 and CA72-4 appeared to improve classification beyond cytology alone [18]. CRP has been proposed as a practical adjunct, with studies suggesting its value in distinguishing malignant from benign effusions [13,52]. In surgical cohorts, peritoneal CRP has even been explored as a predictor of postoperative complications such as anastomotic leakage [34].

Our proposed diagnostic algorithm (Figure 6) reflects these scenarios. Cytology remains the first-line diagnostic tool, but when results are negative or uncertain, biomarker analysis may provide additional guidance. In practice, this could mean supporting earlier recognition of peritoneal metastasis in gastric cancer, reducing repeated aspirations in ovarian cancer or even helping to distinguish malignancy from tuberculosis in atypical ascites.

At the same time, biomarker cutoff values and assay thresholds varied substantially across studies; therefore, no universally accepted decision points have been validated. For this reason, the algorithm should be regarded as a conceptual framework rather than a prescriptive tool, with application tailored to assay-specific characteristics and local protocols.

Emerging markers such as VCAM1, CD146 and CYFRA 21-1 remain exploratory but underline the need for prospective multicenter validation in real-world cohorts before biomarker-guided approaches can be confidently adopted in clinical practice.

### 4.4. Strengths and Limitations

This review followed the PRISMA guidelines and included a broad range of studies with diverse geographic origins and methodological approaches, which strengthened its external validity. Data extraction was systematic and categorized by fluid type, marker category and diagnostic performance. Both established and emerging biomarkers were evaluated to reflect the current and evolving field of ascitic fluid diagnostics.

However, the included studies differed significantly in design, marker thresholds and outcome reporting. Several studies did not report complete diagnostic metrics, and fluid sampling protocols varied. The use of peritoneal washings and lavage fluid may have introduced variability, although these methods reflect real-world clinical practice. Heterogeneity in study designs, assay methods, cutoff values, patient demographics and laboratory practices reduced comparability and may have influenced diagnostic accuracy. A structured risk-of-bias assessment was conducted using ROBINS-I, which identified recurring limitations. Most studies were rated as having some concerns, mainly due to the lack of blinding of patients and personnel, the retrospective design of many cohorts, variability in cutoff determination and incomplete follow-up. In many cases, blinding was not possible, as both patients and clinicians were aware of underlying diagnoses and the presence of ascites. Furthermore, small sample sizes and single-center recruitment were common, while only a few studies were multicenter or prospective. A more detailed interpretation of common bias domains, including limitations in blinding, retrospective design and threshold variability, is provided in Section 2.2.

The literature search was limited to PubMed and Scopus; while these cover the majority of biomedical research, the exclusion of databases such as Embase and Web of Science may have led to omission of additional relevant studies. Additionally, a formal meta-analysis could not be performed because of methodological heterogeneity; therefore, conclusions are based on a narrative synthesis of study-level findings.

### 4.5. Research Gaps and Future Directions

Several gaps identified in this review warrant further investigation. Although markers such as microRNAs, autotaxin and soluble PD-L1 demonstrate promising diagnostic performance in early studies, their use remains investigational and is not yet validated for routine clinical application. Standardized protocols for sample collection, storage and analysis were also needed. Second, very few studies compared different fluid types within the same group of patients. Future research should explore whether peritoneal or lavage fluids, when combined with serum markers, could reliably replace ascitic fluid when it is not available. Third, evidence on the use of ascitic biomarkers for longitudinal monitoring or treatment response remained limited. This represents a critical area for future research. Finally, malignancies such as hepatocellular carcinoma, gastric cancer and rare gynecological tumors were underrepresented in the current literature and require more focused investigation.

Future studies should prospectively validate the proposed diagnostic algorithm in larger, multicenter cohorts using standardized assay platforms and predefined biomarker cutoffs. Such validation is essential to confirm its reproducibility and clinical utility before integration into routine practice.

## 5. Conclusions

This review highlighted that tumor and inflammatory markers in ascitic and related peritoneal fluids provided meaningful diagnostic and prognostic value. Markers such as CEA, CA125, IL-6 and VEGF consistently performed well, most notably when used in panels or as ratios with serum values. Importantly, ascitic fluid testing proved helpful in situations where cytology was inconclusive, or when serum markers alone lacked sensitivity, offering an earlier path to diagnosis and a clearer guide for patient management.

For clinicians, these findings highlight that ascitic fluid biomarkers can complement established tools rather than replace them. By capturing local tumor activity and inflammatory changes directly within the peritoneal cavity, these markers provide information that serum testing may miss. This added layer of insight can support more confident treatment decisions, particularly in patients with suspected gastrointestinal or ovarian malignancies.

Looking ahead, large prospective and multicenter studies are still needed to validate the clinical utility of both established and emerging biomarkers. Molecular and proteomic approaches show promise, but they remain at an early stage. Integrating ascitic fluid biomarkers into diagnostic pathways represents an important opportunity to connect laboratory medicine more directly with clinical care, with the ultimate goal of improving outcomes for patients with unexplained ascites.

In summary, this review demonstrates that ascitic fluid biomarkers not only complement cytology but also open avenues for earlier, more accurate diagnosis and better-targeted treatment strategies. Their integration into diagnostic pathways could help reduce delays in management, particularly in oncology and infection, where timely decisions are critical. Future work should prioritize prospective, multicenter validation to confirm their utility and establish standardized diagnostic thresholds that will allow translation into routine clinical practice.

## Figures and Tables

**Table 1:** Baseline characteristics of studies included in the review.

Study	Sample Size	Age	Study Duration	Study Center	Study Type
Abdel-Razik et al., 2016 [13]	315	Benign: 55.4 ± 15; Malignant: 53.9 ± 16	May 2013–July 2015	Mansoura University, Egypt	Prospective
Bérgamo et al., 2023 [16]	118	Mean 68 years (42–92)	Not specified	Althaia Xarxa Assistencial Universitària de Manresa, Manresa, Catalonia, Spain	Retrospective observational
Castro et al., 2015 [17]	30 ascites samples (20 cancer, 10 benign)	Not specified	Not specified	Massachusetts General Hospital Cancer Center, USA	Proof-of-concept nanotechnology platform
Choi et al., 2023 [18]	138	Not specified	Not specified	Gangnam Severance Hospital, Yonsei University, South Korea	Prospective observational
Chudecka-Głaz et al., 2014 [19]	139	24–88 years	2012–2013	Central Laboratory of the Independent Public Hospital, Poland	Prospective observational
Du et al., 2022 [20]	169	TBP: Median 44; PC: Median 60	May 2015–January 2022	Union Hospital, Tongji Med. College, China	Retrospective cohort
Faria et al., 2019 [21]	120 (33 peritoneal, 87 pleural)	Mean 57.7 ± 16.6	Not specified	Hospital das Clínicas, Univ. of São Paulo, Brazil	Prospective observational
Han et al., 2018 [22]	177 (104 malignant, 73 benign)	Mean 56 ± 13 (20–80)	January 2010–December 2014	Union Hospital, Tongji Med. College, China	Retrospective observational
Hrvat et al., 2024 [23]	28 malignant, 1 benign ascites; healthy serum	Not specified	Not specified	Univ. Hospitals Essen & Cologne, Germany	In vitro mechanistic coculture study
Jain et al., 2022 [24]	93	Mean 47 (14–81)	July 2018–December 2019	PGI Medical Education & Research, Chandigarh, India	Prospective observational
Janež et al., 2022 [25]	43	Mean 69.9 ± 13.6	Not specified	Univ. Medical Centre Ljubljana, Slovenia	Prospective pilot cohort
Jerman et al., 2014 [26]	33	Mean 43 ± 1.82	December 2011–September 2013	University Medical Centre Ljubljana, Slovenia	Prospective protocol development
Jin et al., 2018 [27]	85 GC, 27 benign; ELISA: 57 GC + 27 benign	Not specified	Not specified	Seoul National Univ. College of Medicine & Hospital, South Korea	Proteomics discovery + ELISA validation
Kim et al., 2019 [28]	495	Median 65 (24–93)	January 2006–November 2014	St. Mary’s Hospital, Catholic Univ. of Korea, South Korea	Retrospective cohort
Kocbek et al., 2015 [29]	98 (58 endo, 40 controls)	Not stated	Not specified	Univ. of Ljubljana & Berne, Slovenia and Switzerland	Prospective
Kolomeyevskaya et al., 2015 [30]	39	Median 61 (range 44–82)	Not specified	Roswell Park Cancer Institute, Buffalo, NY, USA	Prospective observational cohort
Liang et al., 2021 [31]	512	Median 58 (IQR 49–64.5)	May 2009–October 2019	Aerospace Center Hospital, Beijing, China	Prospective observational
Liu et al., 2014 [6]	437	Not specified	2007–2012	Union & Tongji Hospitals + Qingdao Univ., China	Retrospective observational
Liu et al., 2024 [32]	418	Not specified	June 2018–October 2023	Union Hospital, Tongji Med. College, Wuhan, China	Retrospective cohort
Masoumi-Moghaddam et al., 2015 [33]	98	Median 62 (35–84)	2001–2012	St. George Hospital, Sydney, Australia	Retrospective
Mužina Mišić et al., 2023 [34]	59	Median 68 (range 25–91)	January–October 2019	Sestre Milosrdnice University Hospital Center, Croatia	Prospective observational
Nakul-Kadam et al., 2016 [35]	100 (50 SBP, 50 sterile ascites)	Mean 46.84 ± 12.46	September 2013–October 2015	Jawaharlal Nehru Medical College, Wardha, India	Prospective case-control
Okła et al., 2018 [36]	101	Median 56 (20–89)	2012–2016	Medical University of Lublin, Poland	Prospective observational
Prunoiu et al., 2020 [37]	100	Adults (not specified)	May 2016–December 2017	Bucharest Oncology Institute, Romania	Prospective observational
Schindler et al., 2018 [38]	45 patients (15 PCA, 15 SBP, 15 PH)	Mean 61.6 ± 10.4 years	Not specified	Univ. of Magdeburg; Lithuanian Univ. of Health Sciences, Germany, Lithuania	Prospective molecular profiling
Song et al., 2018 [39]	259 (82 CRC, 177 benign)	Median 60 (range 17–87)	January 2000–May 2013	Kosin University, Busan, South Korea	Retrospective observational
Sparreboom et al., 2020 [40]	292	Median 63 (IQR 57–71)	August 2015–October 2017	10 hospitals in Netherlands and Belgium	Prospective multicenter cohort
Sun et al., 2017 [41]	64 malignant, 30 benign ascites	Mean 56.04 ± 14.53 years	January–December 2016	First Affiliated Hospital of Xi’an Medical University, China	Prospective diagnostic biomarker study
Sun et al., 2024 [42]	35 HCC ascites, 35 benign	HCC: 62.3 ± 9.1; Benign 55.3 ± 9.8	September 2022–March 2024	Second Affiliated Hospital of Chongqing Med. Univ., China	Prospective diagnostic & mechanistic study
Tong et al., 2017 [43]	292 (TBP: 101; non-OCA: 120; OCA: 71)	TBP: 40.8 ± 18.4; non-OCA: 61.9 ± 12.8; OCA: 56.3 ± 12.4	January 2009–December 2013	West China Hospital, Sichuan University, China	Retrospective observational
Trapé et al., 2020 [44]	157	Mean 67.7 (35–96)	January 2008–December 2017	Althaia Xarxa Assistencial Universitària de Manresa, Manresa, Catalonia, Spain	Retrospective observational
Uehara et al., 2020 [45]	81 patients with PC and ascites	Median 64 (range 28–87)	2010–2018	National Cancer Center Hospital, Tokyo, Japan	Retrospective diagnostic accuracy study
V. Dalal et al., 2018 [46]	45 (30 epithelial ovarian cancer (EOC) patients; 15 benign controls)	Patients: Mean 51.63 ± 12 years; Controls: Mean 48.40 ± 5.05 years	Not specified	All India Institute of Medical Sciences (AIIMS), New Delhi, India	Prospective observational
Wang et al., 2023 [47]	281 (183 PMP + 98 controls)	Median 56 (19–77)	May 2012–June 2020	Aerospace Center Hospital, Beijing, China	Retrospective cohort
Yamamoto et al., 2014 [48]	193	Mean 68.9 ± SD	October 2006–March 2011	Hiroshima Atomic Bomb Survivors Hospital, Japan	Prospective observational
Yamamoto et al., 2018 [49]	8 OC ascites; 10 benign peritoneal fluids	OC: Mean 64 ± 12 years	Not specified	Brigham & Women’s Hospital; Tufts Medical Center, USA	Exploratory observational with qPCR & RNA-seq
Yang and Zhang, 2024 [50]	22 (12 GC malignant, 10 benign ascites)	Matched (not numerically specified)	Not specified	Sunshine Union Hospital, Weifang, China	Bioinformatic analysis using GEO/TCGA
Yu et al., 2021 [51]	63	TB: Mean 43.6 ± 18.5; Malignant: 60.3 ± 12.3	November 2013–December 2017	First Affiliated Hospital of Nanchang Univ., China	Prospective observational
Yüksel et al., 2014 [52]	91	Adults with ascites	February–November 2013	Dıskapı Yıldırım Beyazıt Hospital, Turkey	Prospective
Záveský et al., 2019 [53]	60 (26 OC patients, 34 controls)	Median 60 (ascites), 62 (lavage)	Not specified	General Univ. Hospital Prague; Univ. Hospital Brno, Czech Republic	Prospective observational
Zhan et al., 2016 [54]	1012	Mean ~52	2007–2012	Renmin Hospital of Wuhan University, China	Retrospective observational
Zhu et al., 2015 [55]	76 (45 malignant, 31 benign)	Malignant: 54.4 ± 11.8; Benign: 42.7 ± 16.4	June 2012–June 2014	People’s Hospital Anqing & Zhejiang Prov. Hospital, China	Prospective observational

Abbreviations: AIIMS: All India Institute of Medical Sciences; CRC: colorectal cancer; ELISA: enzyme-linked immunosorbent assay; EOC: epithelial ovarian cancer; GC: gastric cancer; GEO: Gene Expression Omnibus; IQR: interquartile range; OC: ovarian cancer; OCA: ovarian cancer ascites; PC: peritoneal carcinomatosis; PCA: principal component analysis; PGI: Postgraduate Institute of Medical Education and Research; PH: portal hypertension; PMP: pseudomyxoma peritonei; RNA: ribonucleic acid; SBP: spontaneous bacterial peritonitis; TB: tuberculosis; TBP: tuberculous peritonitis; TCGA: The Cancer Genome Atlas.

**Table 2:** Tumor marker evaluation and diagnostic utility.

Marker(s)	Fluid(s) Measured	Cancer(s)	Marker Used Alone or in Panel	Diagnostic and Prognostic Value
**CEA**	Ascitic fluid	Ovarian/Mixed malignancies	CEA + CA125 [43]; CEA + CA125 ± PET/CT [22]	**Diagnostic Value:** composite index (CEA × CA125) AUC = 0.946, higher than either marker alone, distinguishing OCA from TBP/non-OCA [43]. PET/CT combined with ascitic CEA + CA125 improved diagnostic accuracy [22]. **Prognostic Value:** Not specified.
		Pseudomyxoma peritonei (PMP)	CEA in marker count [31]; CEA + CA125 + CA19-9 [47]	**Diagnostic Value:** No AUC reported. Both studies used multi-marker counts/panels rather than single-marker diagnostics. **Prognostic value:** Higher number of positive markers associated with higher pathology grade and poorer survival [31]. Multi-marker positivity stratified survival [47].
		Colorectal	CEA alone [28,39]	**Diagnostic Value:** ascitic CEA had high accuracy (AUC 0.996) and was the best-performing single marker [39]. CEA ≥ 5 ng/mL indicated higher risk factors [28]. **Prognostic Value:** Yes [28].
	Ascitic + serum	Mixed malignancies/Multicancer cohorts [44,51,55]	CEA + CA125 + CA19-9 [51]; CEA + CA15-3 + CA72-4 + CA19-9 [44]; CEA + CA125 + CA19-9 [55]	**Diagnostic Value:** Fisher model with ascites/serum ratios achieved AUC = 0.908, outperforming single markers [51]. F/S strategy improved accuracy (sensitivity ~76%, specificity ~95–99%) [44]. CEA in ascites (AUC = 0.859) and CEA ascites/serum ratio (AUC = 0.879) showed high specificity; panels increased sensitivity [55]. **Prognostic Value:** not specified.
	Peritoneal/Pleural	Mixed malignancies [21,48]; Non-malignant (AL context) [25]	CY-CEA + CY-CA 72-4 [48]; CEA ± NGAL [21]; CEA ± Lactate [25]	**Diagnostic Value:** CY-CEA/CY-CA72-4 positivity predicted peritoneal dissemination [48]. CEA + NGAL improved diagnostic performance (accuracy 79.2%) for malignant effusions vs. single markers [21] Not cancer-related, used perioperatively to detect anastomotic leakage [25]. **Prognostic Value:** combined positivity associated with worse 5-year survival [48].
**CA 125**	Serum + Ascitic fluid	Mixed malignancies	Alone and in panel [55]; alone and in ascitic-to-serum ratio [51]	**Diagnostic Value:** ascitic CA125 AUC = 0.706; ascitic-to-serum ratio AUC = 0.726 [55]; AUC = 0.823 [51]. Limited standalone value; ratios and panels improved discrimination. **Prognostic Value:** not specified.
**CA 19-9**	Ascitic fluid	Peritoneal carcinomatosis (PC) vs. tuberculous peritonitis (TBP)	Alone and in panel [24]	**Diagnostic Value:** In combination with CEA and CA72-4, achieved sensitivity ~86% and specificity ~55% [24]. **Prognostic Value:** not specified.
	Ascitic + Serum	Mixed malignancies	Alone and in panel [6,55]	**Diagnostic Value:** ascitic CA19-9 AUC = 0.697, Fisher model combining CEA + CA125 + CA19-9 achieved AUC = 0.984 [55]. The combination panel (CEA + CA19-9 + CA15-3) improved to sensitivity 85%, specificity 97% [6]. **Prognostic Value:** not specified.
		Malignant vs. benign ascites	Alone and in ascitic-to-serum ratio [55]	**Diagnostic Value:** ascites AUC 0.697, the ascitic-to-serum ratio clearly improved discrimination, being highly diagnostic. **Prognostic Value:** not specified.
		Malignant vs. tuberculous	Alone and in combination [20]	**Diagnostic Value:** ascitic CA19-9 sensitivity = 56% and specificity = 88% [20]. **Prognostic Value:** not specified.
**HE4**	Ascitic fluid	Malignant vs. benign ascites	Single marker [16]	**Diagnostic Value:** sensitivity (21.2%) and high specificity (100%) for malignancy. When combined with adjusted clinical parameters (ADA, CRP, PMN, eGFR), sensitivity improved modestly. **Prognostic Value:** not specified [16].
		Ovarian cancer vs. benign gynecologic diseases	Single marker [19]	**Diagnostic Value:** AUC = 0.575. No significant difference between cancer and benign states; also elevated in benign effusions. **Prognostic Value:** Higher ascitic HE4 associated with worse overall survival and poorer platinum response [19].
**CA 72-4**	Ascitic fluid	Malignant vs. benign ascites	Alone and in panel [24,44]	**Diagnostic Value:** panel (CEA + CA 19-9 + CA 72-4) sensitivity 86%, specificity 55% [24]. Panel (CEA + CA 15-3 + CA 72-4) diagnostic accuracy ~90% [44]. **Prognostic Value:** not specified.
		Gastric cancer	Alone and in combination [48]	**Diagnostic value:** sensitivity 77%, specificity 84.4%; CA 72-4 and CEA in lavage fluid strongly correlated. **Prognostic value:** combined positivity predicted significantly poorer survival [48].
	Ascitic + Serum	Pseudomyxoma peritonei (PMP)	Panel (CEA, CA 125, CA 19-9, CA 72-4, CA 242) [31]	**Diagnostic Value:** panel evaluated in relation to pathology grade and completeness of cytoreduction. **Prognostic Value:** high number of elevated markers significantly associated with poorer survival [31].
	Peritoneal lavage	Gastric cancer	Alone, in panel with CEA [48]	**Diagnostic Value:** combination with CEA in lavage fluid improved accuracy. **Prognostic Value:** combined CA72-4 and CEA positivity significantly associated with poorer survival [48].
**CYFRA 21-1**	Ascitic fluid	Malignant ascites [16], mixed effusions [44]	Single marker	**Diagnostic Value:** levels increased in malignant vs. benign ascites [16]. Standalone accuracy not consistently reported [44]. **Prognostic Value:** not specified.
**CA 15-3**	Ascitic fluid	Malignant, benign ascites	Alone, in panel and ascitic-to-serum ratio [32,44]	**Diagnostic Value:** Panel (CEA, CA15-3, CA72-4) specificity 100%, sensitivity of 79.7%. In patients with negative cytology, the sensitivity of three markers was 69.7%, at a specificity of 100% [44]. AUC = 0.850 (ascites); ascitic-to-serum ratio AUC = 0.863 [32]. **Prognostic Value:** not specified.
		Peritoneal carcinomatosis (PC) vs. tuberculous peritonitis (TBP)	Alone and in panel [24]	**Diagnostic Value:** in panel (CEA or CA 15-3 or CA 19-9) there is higher accuracy 94.67%. **Prognostic Value:** not specified [24].
		Mixed malignancies	Alone and in panel [6]	**Diagnostic Value:** for CA 15-3 alone: sensitivity 23%, specificity 100%. Panel (CEA + CA 19-9 + CA 15-3): sensitivity 85.87%, specificity 97.32%. **Prognostic Value:** not specified
	Ascitic + Serum	Pseudomyxoma peritonei (PMP)	Panel [31,47]	**Diagnostic Value:** CA 15-3 included in multi-marker count/panel for staging and assessment of completeness of cytoreduction. **Prognostic Value:** elevated positive markers (including CA 15-3) associated with poorer survival [31,47].
**AFP**	Ascitic fluid	Mixed malignancies	Panel [55]	**Diagnostic Value:** AFP measured in combination with CA125 and CA19-9 in diagnostic panels, including evaluation of ascitic-to-serum ratios. **Prognostic Value:** not specified [55].
**Autotaxin (ATX)**	Ascitic fluid	EOC (Epithelial ovarian cancer)	Alone and in panel with CA125 [18]	**Diagnostic Value:** elevated in malignant ascites compared to benign; promotes cancer invasion and stemness. **Prognostic Value:** high ATX linked to advanced FICO stage and shorter disease-free survival [18].
**miRNA panel**	Ascitic fluid	Ovarian cancer	miR-21, miR-23b, miR-29a [53]; miRNA panel in EVs [49]; miR-21/miR-223 ratio [38]	**Diagnostic Value:** miRNA levels significantly elevated in tumor-cell–enriched effusions compared with benign [53]. EV-derived miRNAs enabled tumor cell classification; provided added discriminatory power [49]. Very high discriminatory power between malignant and benign effusions [38]. **Prognostic Value:** expression levels of miR-21, miR-23b and miR-29a linked to poor progression-free and overall survival [53].
**MSLN**	Peritoneal fluid	EOC (epithelial ovarian cancer)	Single marker [36]	**Diagnostic Value:** PF MSLN correlated with plasma MSLN. **Prognostic Value:** not specified for peritoneal fluid; only plasma MSLN was associated with prognosis [36].

Abbreviations: ADA: adenosine deaminase; AL: anastomotic leakage; ATX: autotaxin; CY: cytology; CYFRA: cytokeratin 19 fragment; DFS: disease-free survival; EOC: epithelial ovarian cancer; GI: gastrointestinal; IU: international units; MSLN: mesothelin; NGAL: neutrophil gelatinase-associated lipocalin; OS: overall survival; PC: peritoneal carcinomatosis; PF: peritoneal fluid; PFS: progression-free survival; PMN: polymorphonuclear neutrophils; PMP: pseudomyxoma peritonei; TBP: tuberculous peritonitis.

**Table 3:** Inflammatory marker evaluation and diagnostic utility.

Marker(s)	Fluid(s) Measured	Cancer(s)	Marker Used Alone or in Panel	Diagnostic and Prognostic Value
**CRP**	Ascitic fluid + Serum	HCC, CRC, Ovarian [13]	Alone and in Panel	**Diagnostic Value:** ascites AUC = 0.842, serum AUC = 0.821. The combined model yielded best performance. **Prognostic Value:** VEGF correlated with stage; CRP with severity [13].
		Malignant ascites	Alone [16]	**Diagnostic Value:** ascitic fluid CRP has stronger performance than serum. **Prognostic Value:** not specified [16].
	Ascitic fluid	Cirrhosis/Non-malignant	Alone [35]	**Diagnostic Value:** higher level of hs-CRP before antibiotic therapy of the patients with SBP than that of the patients without SBP. **Prognostic Value:** greater ascitic fluid hs-CRP levels in SBP poorly correlate with the prognosis of the patients with cirrhosis [35].
	Peritoneal fluid + Serum	Colorectal	Alone [34]	**Diagnostic Value:** peritoneal fluid AUC = 0.833 (POD4), serum AUC = 0.886 (POD4). Peritoneal fluid slightly less accurate than serum. Prognostic **Value:** CRP POD4 predicted AL [34].
**IL-6**	Ascitic fluid	Ovarian (EOC)	Alone and in Panel [30,46]	**Diagnostic Value:** AUC = 1.0 [46]. Diagnostic performance enhanced in combination with TNF-α [30]. Prognostic **Value:** Shorter PFS and OS [30]. IL-6 and VEGF-A predicted PFS [46].
			Panel (IL-6 + TNF-α) [30]	**Diagnostic Value:** Best recurrence stratification. Prognostic **Value:** shorter PFS and OS [30].
			Panel (IL-6 + VEGF-A) [46]	**Diagnostic Value:** both AUC = 1.000. Combined use showed strongest performance. Prognostic **Value:** IL-6 and VEGF-A predicted PFS [46].
	Tumor tissue (IHC)	Ovarian (EOC)	Alone [33]	**Diagnostic Value:** Tumoral IL-6 predicted post-treatment ascites. Prognostic **Value:** IL-6 expression was predictive of the occurrence of post-treatment ascites in ovarian cancer patients [33].
**VEGF**	Ascitic fluid	Ovarian (EOC), Gastric, Colorectal, HCC	Alone and in Panel [46,54]	**Diagnostic Value:** Strong prognostic marker for EOC [46]. High VEGF = shorter OS [54]. **Prognostic Value:** high VEGF predicted shorter OS [54].
	Ascitic + Serum	HCC, CRC, Ovarian	Alone and in Panel (VEGF/VEGF-A) [13]	**Diagnostic Value:** highest discriminative performance. **Prognostic Value:** VEGF correlated with stage [13].
**sPD-L1**	Ascitic + Serum	Pleural and Peritoneal effusions	Alone [21]	**Diagnostic Value:** sensitivity 52.2%, specificity 58.5% in the diagnosis of malignant effusions. Prognostic **Value:** not specified [21].
**TNF-α**	Ascitic	Epithelial Ovarian Cancer (EOC)	Alone and in Panel [30]	**Diagnostic Value:** shorter PFS and OS. **Prognostic Value:** TNF-α levels were predictive of survival outcomes [30].
**sOPN, Scd44-V6, sVCAM-1**	PF, Washing fluid	Benign gynecologic disease	Panel [26]	**Diagnostic Value:** sOPN—reproductible in washing samples; sCD44-v6—strong correlation between fluid types; sVCAM-1—validated washing as PF substitute. **Prognostic Value:** not specified [26].
**MMP-9**	Drain fluid	Colorectal	Alone and in Panel [40]	**Diagnostic Value:** best predictor of AL when combined with CRP. **Prognostic Value:** CRP + MMP-9 levels on day 3 predicted AL [40].
**Leukocyte count**	Peritoneal fluid	Colorectal	Alone [37]	**Diagnostic Value:** best when used with CRP. **Prognostic Value:** CRP and leukocyte count predicted AL [37].

Abbreviations: AL: anastomotic leakage; CRC: colorectal cancer; EOC: epithelial ovarian cancer; IHC: immunohistochemistry; MMP: matrix metalloproteinase; OS: overall survival; PF: peritoneal fluid; PFS: progression-free survival; SBP: spontaneous bacterial peritonitis; VEGF-A: vascular endothelial growth factor-A.

## Data Availability

No new data were created or analyzed in this study. Data sharing is not applicable to this article. The PRISMA 2020 checklist used for reporting compliance is available.

## Abbreviations

The following abbreviations are used in this manuscript:

Abbreviation	Definition
PRISMA	Preferred Reporting Items for Systematic Reviews and Meta-Analyses
ROBINS-I	Risk Of Bias In Non-randomized Studies—of Interventions
VEGF	Vascular endothelial growth factor
VEGF-A	Vascular endothelial growth factor A
LDH	Lactate dehydrogenase
CRP	C-reactive protein
hs-CRP	High-sensitivity C-reactive protein
PD-L1	Programmed Death-Ligand 1
CT	Computed Tomography
PET	Positron Emission Tomography
FDG	Fluorodeoxyglucose
ROC	Receiver Operating Characteristic
RNA	Ribonucleic Acid
RT-PCR	Reverse transcription polymerase chain reaction
HCC	Hepatocellular carcinoma
EV	Extracellular vesicles
CRC	Colorectal cancer

## Appendix A

### Search Strategy and Keywords Used

This systematic review included studies published between January 2014 and December 2024. The last search update was performed in May 2025. Searches were conducted in the following databases: PubMed and Scopus. Reference lists of included studies were also screened to identify additional relevant literature.

Inclusion Filters Applied: Human studies, English language, Measurement of markers in ascitic/peritoneal fluid

Search Terms Used (combined with Boolean operators “AND”/“OR”):

General Terms: “ascitic fluid”, “peritoneal fluid”, “diagnosis”, “prognosis”, “malignant ascites”, “infectious ascites”, “inflammatory ascites”

Tumor Markers: “tumor markers”, “CEA”, “CA125”, “CA19-9”, “CA15-3”, “HE4”, “Autotaxin”

Inflammatory Markers: “CRP”, “hs-CRP”, “VEGF”, “IL-6”, “IL-8”, “TNF-α”, “MMP-9”

Novel Biomarkers: “microRNA”, “miRNA”, “miR-200”, “miR-223”, “miR-21”, “miR-141”, “miR-429”, “sHLA-G”, “Periostin”, “exosomes”, “glycoproteins”, “NETs”, “extracellular vesicle (EV) proteins”

Example Search Combinations:

“ascitic fluid” AND “CEA”

“peritoneal fluid” AND “VEGF”

“ascitic fluid” AND “tumor markers”

“malignant ascites” AND “CRP”

These combinations were iteratively adapted to capture relevant literature on tumor, inflammatory and other biomarkers evaluated in ascitic samples.
